# Oct4 activates IL-17A to orchestrate M2 macrophage polarization and cervical cancer metastasis

**DOI:** 10.1007/s00262-023-03596-z

**Published:** 2024-03-02

**Authors:** Zhuoqiong Bian, Xiaoling Wu, Qing Chen, Qing Gao, Xiang Xue, Yidong Wang

**Affiliations:** 1 Department of the Fifth Rheumatology, The Fifth Hospital of Xi’an City, Xi’an, 710000 Shaanxi China; 2 Department of Obstetrics and Gynecology, Second Affiliated Hospital, Xi’an Jiao Tong University, 157 West Fifth Road, Xincheng District, Xi’an, 710000 Shaanxi China

**Keywords:** Cervical cancer, IL-17A, Oct4, M2 macrophage, p38 signaling

## Abstract

**Background:**

Cervical cancer is a common malignant tumor in the female. Interleukin (IL)-17A is a proinflammatory factor and exerts a vital function in inflammatory diseases and cancers. M2 macrophage has been confirmed to promote tumor development. Nevertheless, it is not yet known whether IL-17A facilitates cervical cancer development by inducing M2 macrophage polarization. Therefore, this study was conducted to investigate the regulatory effect of IL-17A on M2 macrophage polarization and the underlying mechanism in cervical cancer development.

**Methods:**

RT-qPCR was utilized for testing IL-17A expression in cancer tissues and cells. Flow cytometry was applied to evaluate the M1 or M2 macrophage polarization. Cell proliferative, migratory, and invasive capabilities were measured through colony formation and transwell assays. ChIP and luciferase reporter assays were applied to determine the interaction between IL-17A and octamer-binding transcription factor 4 (OCT4).

**Results:**

IL-17A expression and concentration were high in metastatic tissues and cells of cervical cancer. IL-17A was found to facilitate M2 macrophage polarization in cervical cancer. Furthermore, IL-17A facilitated the macrophage-mediated promotion of cervical cancer cell proliferative, migratory, and invasive capabilities. Mechanistic assays manifested that Oct4 binds to and transcriptionally activated IL-17A in cervical cancer cells. Furthermore, Oct4 promoted cervical cancer cell malignant phenotype and M2 macrophage polarization by activating the p38 pathway that, in turn, upregulated IL-17A. Additionally, in vivo experiments confirmed that Oct4 knockdown reduced tumor growth and metastasis.

**Conclusion:**

Oct4 triggers IL-17A to facilitate the polarization of M2 macrophages, which promotes cervical cancer cell metastasis.

**Supplementary Information:**

The online version contains supplementary material available at 10.1007/s00262-023-03596-z.

## Introduction

Cervical cancer is one of the most common malignant tumors in the female reproductive system [1]. With the popularization of cervical cancer screening and vaccination, the incidence rate of cervical cancer has gradually declined [2]. Nevertheless, because of the deficiency of obvious early symptoms of cervical cancer, most patients are diagnosed with advanced stage. Therefore, many women still die from cervical cancer every year. At present, the main therapeutic strategies for treating cervical cancer included surgical resection, chemotherapy, and radiotherapy [3]. These methods have effectively improved the prognosis of early cervical cancer patients, but the results for late-stage patients are not satisfactory. Therefore, it is necessary to have a deeper understanding of cervical cancer pathogenesis.

Tumor-associated macrophages (TAM) in the tumor microenvironment (TME) have been confirmed to play an important role in modulating tumor metastasis, angiogenesis and are closely associated with the poor prognosis [4]. TAM can be polarized into two types in accordance with environmental stimulation, namely M1 type and M2 type [5, 6]. M1 polarized macrophages mainly play a pro-inflammatory role in killing tumor cells by highly expressing CD80 and CD86 molecule while secreting elevated levels of IL-1β and TNF-α proinflammatory cytokines. In contrast, M2 polarized macrophages exert anti-inflammatory and tumor promoting effects, by highly expressing CD206 and CD163 molecules and secreting IL-10 anti-inflammatory cytokine [7]. Macrophages can exhibit different phenotypes at multiple stages of tumor progression. In the early stages of tumor development, M1 macrophages exert the leading function in inhibiting angiogenesis and activating immunity to suppress tumor growth [8]. With the development of tumor, macrophages gradually switch towards M2 phenotype and become the dominant type in microenvironment infiltration, accelerating tumor growth [8]. Therefore, improving the understanding of TAM polarization mechanism may contribute to the effective treatment of cervical cancer.

Interleukin (IL)-17A is a proinflammatory factor produced by TH17 cells [9], and it has been verified as the therapeutic target for many autoimmune diseases, such as rheumatoid arthritis [10] and multiple sclerosis [11]. Moreover, the regulatory functions of IL-17A in various cancers have also been confirmed [12]. However, IL-17A functions in tumorigenesis remain controversial. For example, IL-17A has been shown to facilitate cell invasion of lung cancer [13]. IL-17A accelerates colorectal cancer progression via regulating matrix metalloproteinases (MMPs) expression [14]. On the other hand, IL-17A is proved to reduce tumor growth and metastasis, as well as improve prognosis [15]. Importantly, IL-17A-induced polarization of M2 macrophages in various disease models has been reported. For example, IL-17A-stimulated M2 macrophage polarization can facilitate cell migration, angiogenesis, and tumor growth in lung cancer [16]. M2 macrophage activation by IL-17A improves prostate cancer cells' capacity for invasion and metastasis [17]. In oral cancer cells, IL-17A induces M2 macrophage polarization to advance tumor development [18]. IL-17A promotes M2 polarization to facilitate endometriosis progression [19]. Similarly, in another study, it was shown that IL-17A accelerated cervical cancer development by enhancing MMP expression [20] . Nevertheless, it is not yet known whether IL-17A facilitated cervical cancer development via inducing M2 macrophage polarization.

Therefore, the main purpose of this study was to investigate the regulatory effect of IL-17A on M2 macrophage polarization and the underlying mechanism in cervical cancer development.

## Materials and methods

### Patient tissue samples

This study was approved by the Ethics Committee of Second Affiliated Hospital, School of Medicine, Xi’an Jiaotong University. The surgically resected cervical tumor specimens, primary and metastatic tumors and adjacent no-cancerous tissues called para-carcinoma tissues from 30 patients were collected at Second Affiliated Hospital, School of Medicine, Xi’an Jiaotong University between 2018 and 2020. The patients did not receive any radiotherapy or chemotherapy treatment before surgery. Written informed consent was obtained from each participant. After resection, the samples were frozen and stored at −80 °C.

### Cell culture

Cervical cancer cell lines HeLa and SiHa, normal cervical epithelial cell line ende1617, and human monocytic cell line THP-1 all were purchased from ATCC (Manassas, VA). The cells were grown in DMEM media (Gibco, Carlsbad, USA) containing 10% FBS and cultured at 37 ℃ with 5% CO_2_. The 100 ng/ml phorbol 12-myristate 13-acetate (PMA) was utilized for converting THP-1 cells into macrophages for 48 h. HeLa and SiHa cells were plated at 1 × 10^6^ cells per well and treated with 50 ng/mL of IL-17A or PBS as control for 48 h. The treated THP-1 cells were then co-cultured with the treated HeLa and SiHa cells in a HeLa/SiHa: THP-1 (20:1) ratio for the further 48 h, which were considered as THP-1-derived TAMs.

### Cell transfection

To silence Oct4 expression, HeLa and SiHa cells were transfected with 25 nM of Oct4 shRNA (sh-Oct4; Gene-Pharma, Shanghai, China) and the negative control shRNA (sh-NC; Genechem) utilizing Lipofectamine 3000 (Invitrogen, CA, USA) for 48 h.

### Flow cytometry analysis

For analyzing macrophage infiltration into tumor tissues, half of the tumor tissues obtained from injected mice were cut into small pieces and then digested with a collagenase IV/hyaluronidase solution (300 U/mL/200 U/mL) in RPMI 1640 medium at 37 °C for 1–2 h. The cell suspensions were then centrifuged at 350 × *g* for 5 min at 4 °C, followed by labeling of cells with conjugated antibodies against macrophage. The antibodies used for flow cytometry were PE-conjugated CD86 (cat# 555,665) or CD206 (cat# 555,954), FITC-conjugated CD68 (cat# 562,117), and FITC-conjugated CD45 (cat# 340,664). All antibodies were purchased from BD Biosciences, CA, USA. Gating strategy to identify M1 and M2 macrophages is shown in Fig. S1. Additionally, the treated THP-1 cells co-cultured with the treated HeLa and SiHa cells were dyed with PE-conjugated CD86 or CD206 antibodies for half an hour in FACS buffer. Later, cells were subjected to fixation and permeation. After rinsing cells with PBS, cells were dyed with FITC-conjugated CD68 antibody for half an hour. Afterwards, cells rinsed with PBS were subjected to resuspension in FACS buffer, followed by flow cytometry analysis. The flow cytometer (BD Company, USA) was employed for acquiring the cells while macrophage population was measured by using Flow Jo software. CD206, a well-established M2 marker, was used to characterize the M2 macrophage population, and CD86 was used to characterize the M1 macrophage subset.

### RT-qPCR

Total RNA was extracted from tissue specimens or cells using TRIzol reagent (Invitrogen). The cDNA templates were synthesized by a Transcriptor First Strand cDNA Synthesis kit (Takara, Japan). Next, qPCR was performed with SYBR Green II (Takara) using an ABI PRISM 7900 Sequence Detector system (Applied Biosystems, USA). Gene expression was normalized to GAPDH as an internal control, and the values were calculated by 2^−ΔΔCt^ method. The primer sequences used in this study are as follows:

IL-17A-forward 5′-ACTACAACCGATCCACCTCAC-3′

IL-17A-reverse 5′-ACTTTGCCTCCCAGATCACAG-3′

CD206-forward 5′-TCCGGGTGCTGTTCTCCTA-3′

CD206-reverse 5′-CCAGTCTGTTTTTGATGGCACT-3′

CD163-forward 5′-TTTGTCAACTTGAGTCCCTTCAC-3′

CD163-reverse 5′-TCCCGCTACACTTGTTTTCAC-3′

CD86-forward 5′-CTGCTCATCTATACACGGTTACC-3′

CD86-reverse 5′-GGAAACGTCGTACAGTTCTGTG-3′

CD80-forward 5′-AAACTCGCATCTACTGGCAAA-3′

CD80-reverse 5′-GGTTCTTGTACTCGGGCCATA-3′

Oct-4-forward 5′-GCCAGAGGAAAGCACACT-3′

Oct-4-reverse 5′-CAGATCAGCCACARCGC-3′

GAPDH-forward 5′-GAAGGTGAAGGTCGGAGTCA-3′

GAPDH-reverse 5′-GACAAGCTTCCCGTTCTCAG-3′

Tnf-forward 5′- AGAACTCACTGGGGCCTACA -3′

Tnf-reverse 5′- GCTCCGTGTCTCAAGGAAGT -3′

Ros1-forward 5′-GGCTGCCTATGGATTTCTGTG-3′

Ros1-reverse 5′-GCTGCTGGCCCAGATTAGTT-3′

Vegf-forward 5′- TCCTCACACCATTGAAACCA-3′

Vegf-reverse 5′- ATCCTGCCCTGTCTCTCTGT-3′

Tgfβ-forward 5′- GATGTCACCGGAGTTGTGC-3′

Tgfβ-reverse 5′- TGCAGTGTGTTATCCCTGCT-3′

IL12-forward 5′-CCTTGCACTTCTGAAGAGATTGA-3′

IL12-reverse 5′-ACAGGGCCATCATAAAAGAGGT-3′

IL10-forward 5′- CCAAGACCCAGACATCAAGG-3′

IL10-reverse 5′- AAGGCATTCTTCACCTGCTC-3′

Arg1-forward 5′-GTGGAAACTTGCATGGACAAC-3′

Arg1-reverse 5′-AATCCTGGCACATCGGGAATC-3′

Cxcl9-forward 5′- GCAGTGTGGAGTTCGAGGAA -3′

Cxcl9-reverse 5′- AGTCCGGATCTAGGCAGGTT -3′

### ELISA

The supernatant from cells was collected and stored at − 80 ℃ until use. The concentration of IL-17A was estimated by the ELISA kit (CusaBio, Wuhan, China) following the manufacturer’s guidelines.

### Western blot

Cells were lysed with RIPA buffer (Thermo Fisher Scientific, Waltham, MA, USA), and then 20 µg of proteins were separated by 10% SDS-PAGE and transferred onto PVDF membranes. Membranes were blockaded with 5% skim milk, and then cultured with primary antibodies (Abcam, USA) at 4 ℃ for one night. Next day, after washing the membranes 4 times with TBS-T, it was then incubated with the secondary antibody (ab6721, ab6789, Abcam, USA) for additional 2 h. Protein bands were detected using enhanced chemiluminescence (Advansta, Menlo Park, CA, USA), and the data were analyzed with ImageJ software (National Institutes of Health). The specific primary antibodies used were OCT4 (cat# ab181557, Abcam, USA), MMP2 (cat# ab86607, Abcam, USA), MMP9 (cat# ab76003, Abcam, USA), p38 (cat# 8690, Cell Signaling, USA), Phospho-p38 (cat# 9211, Cell Signaling, USA), and IL-17A (cat# ab79056, Abcam, USA). GAPDH (cat# 92,310, Cell Signaling, USA) was used as a loading control.

### Colony formation assay

Cells were seeded into six-well plates (1000 cells/well) and cultured at 37 °C in an incubator. Every third day, the culture medium was changed. After 2 weeks of culture, cells were subjected to fixation with 4% paraformaldehyde for 30 min. Then, cells were stained with 0.1% crystal violet dye (Beyotime) for half an hour. The cell colonies were counted under the microscope.

### Transwell assays

For invasion or migration assays, cells (1 × 10^5^) were suspended in serum-free medium and placed in upper Transwell chambers (Corning, New York, USA) coated with or without Matrigel. Media containing 10% FBS were added to the lower chamber. Following a 24-h incubation period, the migrated cells in the lower chamber were fixed with 4% paraformaldehyde and stained for 15 min with crystal violet dye. The number of migrated and invaded cells was photographed and recorded under the microscope (Olympus, Tokyo, Japan) and counted using ImageJ software.

### Luciferase reporter assay

The wild‐type or mutated Oct4 binding sites to IL-17A promoter were inserted into the pGL3 luciferase reporter vectors (Promega, Madison, WI, USA) to generate pGL3-IL-17A promoter-WT/MUT. Cells were subjected to co-transfection with pGL3-IL-17A promoter-WT/MUT and indicated plasmids for 48 h using the Lipofectamine® 2000 Transfection Reagent (Cat. no. 11668030; Thermo Fisher Scientific, Inc., USA). Finally, a Dual-Luciferase Reporter Assay System (Promega) was employed for testing the luciferase activity.

### ChIP assay

Cells were cross-linked with 1% formaldehyde. Next, lysis buffer was added into the treated cells and chromatin was sheared to DNA fragments of 150–900 bp by sonication. Later, immunoprecipitations of sonicated chromatins were performed using monoclonal anti-Oct4 antibody (ab181557, Abcam, USA) or isotype-matched control IgG Ab (ab172730, Abcam, USA) coupled to microbeads. The precipitated complexes were purified and subjected to amplification via qPCR analysis. The primer sequences for IL-17A used in this study are as follows:

IL-17A promoter forward 5′-GCTACTCGAGGCAAAGCATCTCTGTTCAGC-3′

IL-17A promoter reverse 5′-CGTAAAGCTTGCGTCCTGATCAGCTGGTGC-3′

### Animal experiment

A total of 20 BALB/c nude mice (6 weeks) were obtained from the Vital River Laboratory Animal Technology Co., Ltd. (Beijing, China) and housed under a SPF environment. Animal experiments were approved by the Ethics Committee of Second Affiliated Hospital, School of Medicine, Xi’an Jiaotong University. Mice were separated into the sh-NC group (*n* = 6 mice) and the sh-Oct4 group. HeLa cells (5 × 10^6^) transfected with sh-NC or sh-Oct4 were subcutaneously injected into the back of the mice. After 30 days, mice were euthanized, tumors were dissected, photographed, and weighted. Meanwhile, tumor volume and weight were also measured (*n* = 10 in each group). The tumor volume (mm^3^) was calculated as: tumor volume = length × width^2^ × 0.5. After mice were injected with the HeLa cells (sh-Oct4) for 30 days, they were separated into the sh-Oct4 group and the sh-Oct4 + OCT4-OE group. The sh-Oct4 + OCT4-OE group mice were injected with HeLa cells stably transfected with OCT4-OE vector to observe tumor changes (*n* = 10 in each group). After 15 days, mice were euthanized, and tumors were removed for subsequent analysis.

For the lung metastasis model, 5 × 10^6^ HeLa cells stably transfecting with either sh-NC or sh-Oct4 were injected into the tail vein of mice. After 45 days, the mice were euthanatized. For imaging the tumors, mice were anesthetized and intraperitoneally injected with 30 mg/kg D-luciferin (Sigma-Aldrich, USA) for 10 min, lung metastasis tumor cells labeled by luminescence were imaged through the IVIS Imaging System (PerkinElmer, USA). Pulmonary metastatic nodules were calculated by manually counting individual lesions from metastatic tissue sections in different groups of mice. Thereafter, the data were analyzed using the GraphPad Prism software.

### Immunohistochemical (IHC) analysis

The paraffin-embedded tumor tissues from mice were deparaffinized and rehydrated followed by antigen retrieve and blocking with goat serum. After that, the tissue slices were incubated with the primary antibodies against MMP2 (cat# ab86607, Abcam, USA), MMP9 (cat# ab76003, Abcam, USA), CD206 (cat# ab64693, Abcam, USA), and CD163 (cat# ab182422, Abcam, USA) overnight at 4 ℃. Next day, slices were incubated with secondary antibody (ab6721, ab6789, Abcam, USA) for 1 h. Lastly, the slices were dyed with DAB solution for 5 min. Slices were then observed through the light microscope (Olympus). The number of stained cells was evaluated in 10 random microscopic fields per tissue section, and their average was subsequently calculated. IHC staining of MMP2/MMP9 was calculated by the positive cell numbers per high field. IHC staining of CD206/CD163 was calculated by the positive tumor cell numbers per high field. Image-Pro Plus 6.0 software was utilized for analysis.

### Histological analysis

Tumor tissues were fixed with 4% paraformaldehyde, embedded in paraffin, and cut into 4 μm thickness sections. For assessing histopathological changes, sections were stained with HE and observed under a light microscope (Nikon, Tokyo, Japan).

### Statistical analysis

GraphPad Prism software (version 7.0, USA) was utilized to analyze the data. Data were expressed as means ± SD from three individual repeats. Student’s t test was applied for comparison between two groups. The comparison among multiple groups was analyzed by the one-way ANOVA. Spearman correlation analysis was performed to assess the correlation between Oct4 and IL-17A. *P* < 0.05 represented the statistical difference.

## Results

### IL-17A induces M2 macrophage polarization in cervical cancer

**Fig. 1:**
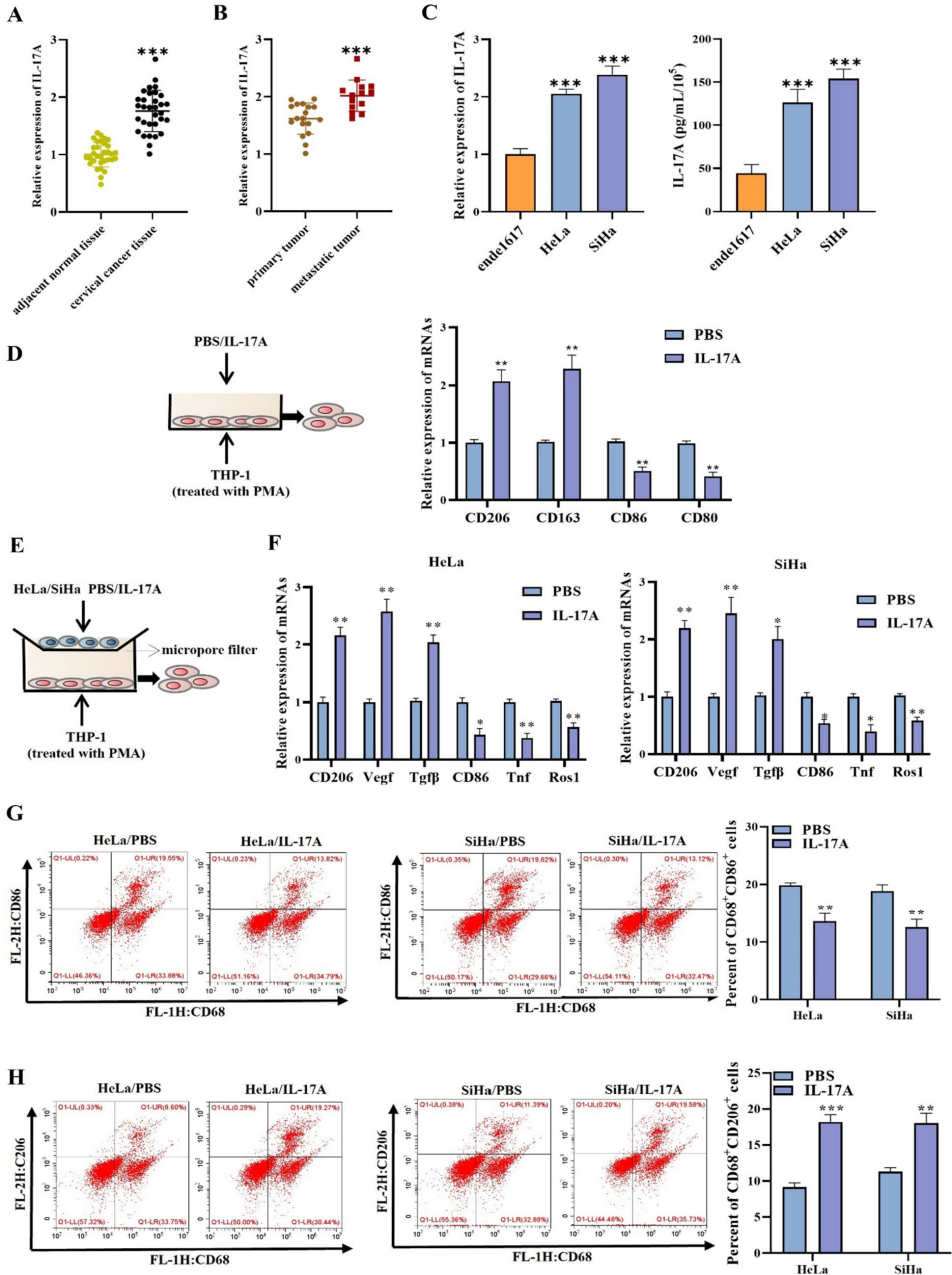
IL-17A induces M2 macrophage polarization in cervical cancer. **A**, **B** RT-qPCR was used to measure IL-17A gene expression level in adjacent normal tissues, cervical cancer tissues, primary tumor tissues, and metastatic tumor tissues. **C** RT-qPCR and ELISA outcomes of IL-17A expression in ende1617, HeLa, and SiHa cells. **D** THP1 cells (treated with PMA) were treated with IL-17A (50 ng/mL) or PBS. RT-qPCR was utilized to measure the gene expression levels of CD206, CD163, CD86, and CD80 expression. **E** The diagrammatic sketch of cell co-culture system. **F** RT-qPCR was utilized to measure the gene expression levels of M1 macrophage markers (CD86, CD80, Tnf1, Ros1) and M2 macrophage markers (CD206, CD163, Veg1, Tgfβ). **G**, **H** Surface expression of CD86 and CD206 was detected in THP-1 (PMA) + Hela(sh-OCT4-1) cells using flow cytometry. The histograms represent the percent of CD86 or CD206 cells in THP-1 (PMA) + Hela(sh-OCT4-1) cells. ^*^p < 0.05; ^**^p < 0.01; ^***^p < 0.001. All experiments were repeated at least three times

Firstly, we detected IL-17A expression in human cervical cancer tissues and human normal adjacent tissues. RT-qPCR illustrated that IL-17A was notably overexpressed in tumor tissues compared to the adjacent normal tissue (Fig. 1A). We further tested IL-17A expression in primary and metastatic tissues of cervical cancer, and the outcomes confirmed the significant overexpression of IL-17A in metastatic tissues (Fig. 1B). In addition, we also observed high IL-17A expression and concentration in HeLa and SiHa cells compared to the normal cervical epithelial cell line ende1617 (Fig. 1C). Previous studies have shown that M2 type TAM can promote cancer metastasis. Therefore, we next tested the impact of IL-17A on macrophage polarization. First, THP1 cells (treated with PMA) were treated with IL-17A (50 ng/mL) or PBS and then explored M1/M2 macrophage subpopulations. Results demonstrated that IL-17A treatment significantly promoted M2 macrophage population in THP1 cells (treated with PMA) as evident from elevated gene expression levels of CD206, and CD163 while downregulated gene expression levels of M1 macrophage markers CD80 and CD86 when compared to the PBS culture (Fig. 1D). We then treated HeLa and SiHa cells with PBS or IL-17A (50 ng/mL) while stimulated monocyte THP-1 cells with PMA. Then the treated HeLa or SiHa cells and PMA-stimulated THP-1 cells were subjected to co-culture (Fig. 1E). Thereafter, M1 macrophage markers (CD86, TNF1, Ros1) and M2 macrophage markers (CD206, Veg1, TGFβ) were detected in the THP-1 (PMA) + Hela/SiHa (IL-17A) cells. In comparison to the PBS treatment, treatment with IL-17-A notably elevated M2 macrophage marker expression levels, while inhibiting M1 macrophage markers levels (Fig. 1F). Similarly, the flow cytometry analysis further determined that IL-17A treatment could reduce the frequency of M1 macrophages while promoting M2 macrophage cell population (Fig. 1G, H). These results confirmed that high expression of IL-17A could facilitate M2 macrophage polarization both in cervical cancer patients and cell lines.

### IL-17A is conducive to macrophage-mediated facilitation of cancer cell proliferation, migration, and invasion

**Fig. 2:**
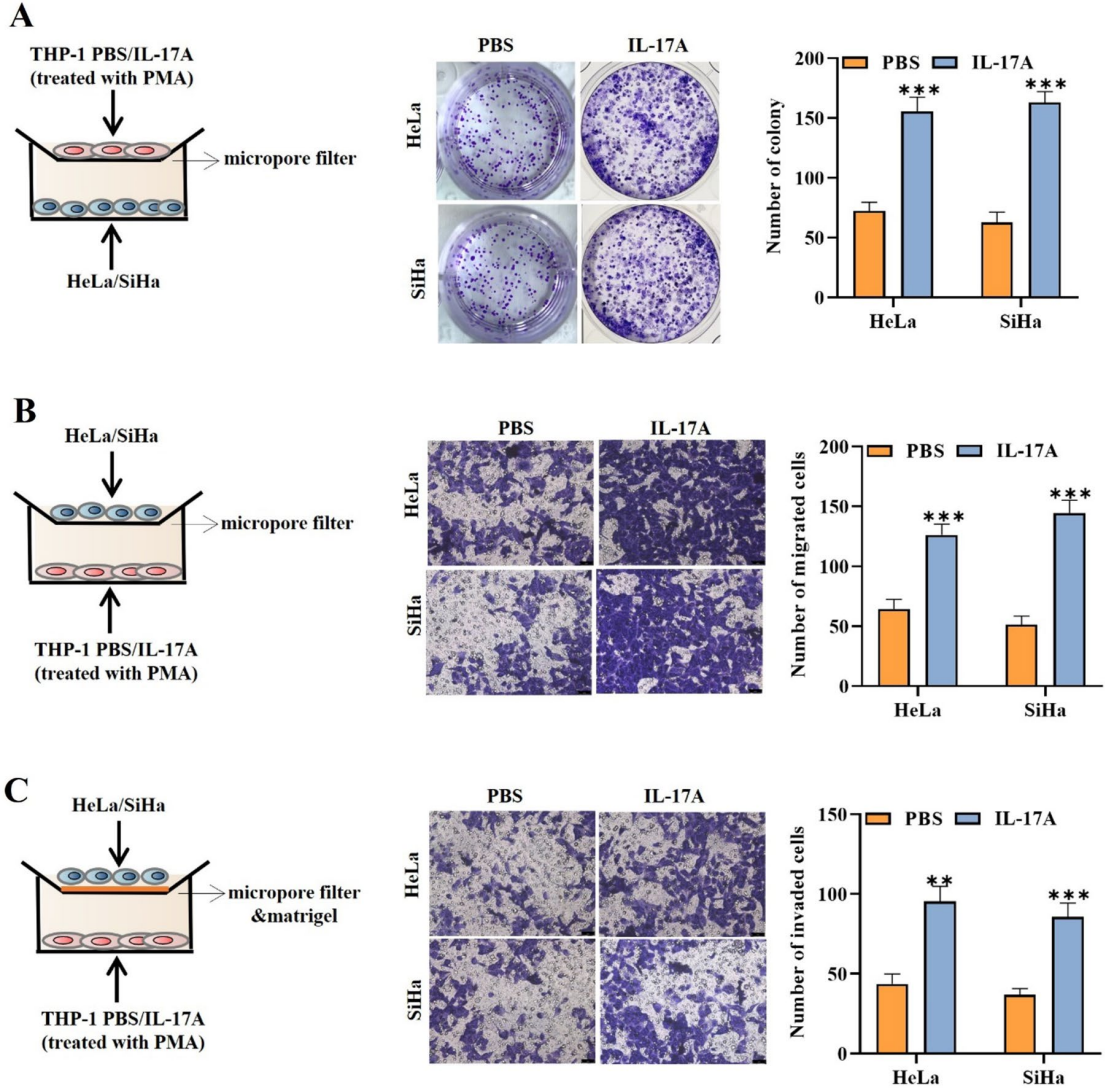
IL-17A secreted by cervical cancer cells is conducive to macrophage-mediated facilitation of cell malignant phenotype. **A**–**C** The proliferative, migratory, and invasive capabilities of HeLa or SiHa cells co-cultured with THP-1-derived TAM cells were evaluated by colony formation **a** and transwell assays **b**–**c** in the PBS treatment group and IL-17A treatment group. ^**^p < 0.01; ^***^p < 0.001. All experiments were repeated at least three times

In the next step, we wanted to investigate whether IL-17A secreted by cervical cancer cells is conducive to macrophage-mediated facilitation of cell malignant phenotype. For this purpose, we co-cultured the PMA-stimulated THP-1 cells with the conditioned medium of cervical cancer cells treated with either PBS or IL-17A, resulting in the induction of M2 type macrophages. Then, the impacts of these macrophages and IL-17A in the malignant phenotype of cancer cells were assessed. Through colony formation assay, we found that the cell proliferative capability of HeLa and SiHa cells was increased in the presence of IL-17A compared to the PBS (Fig. 2A). Moreover, transwell assays further manifested that the cell migratory and invasive capabilities were enhanced by IL-17A presence (Fig. 2B, C). These results confirmed that IL-17A secreted by cervical cancer cells could stimulate macrophage to facilitate cell malignant phenotype.

### High expression of Oct4 promotes the secretion of IL-17A by cervical cancer cells

**Fig. 3:**
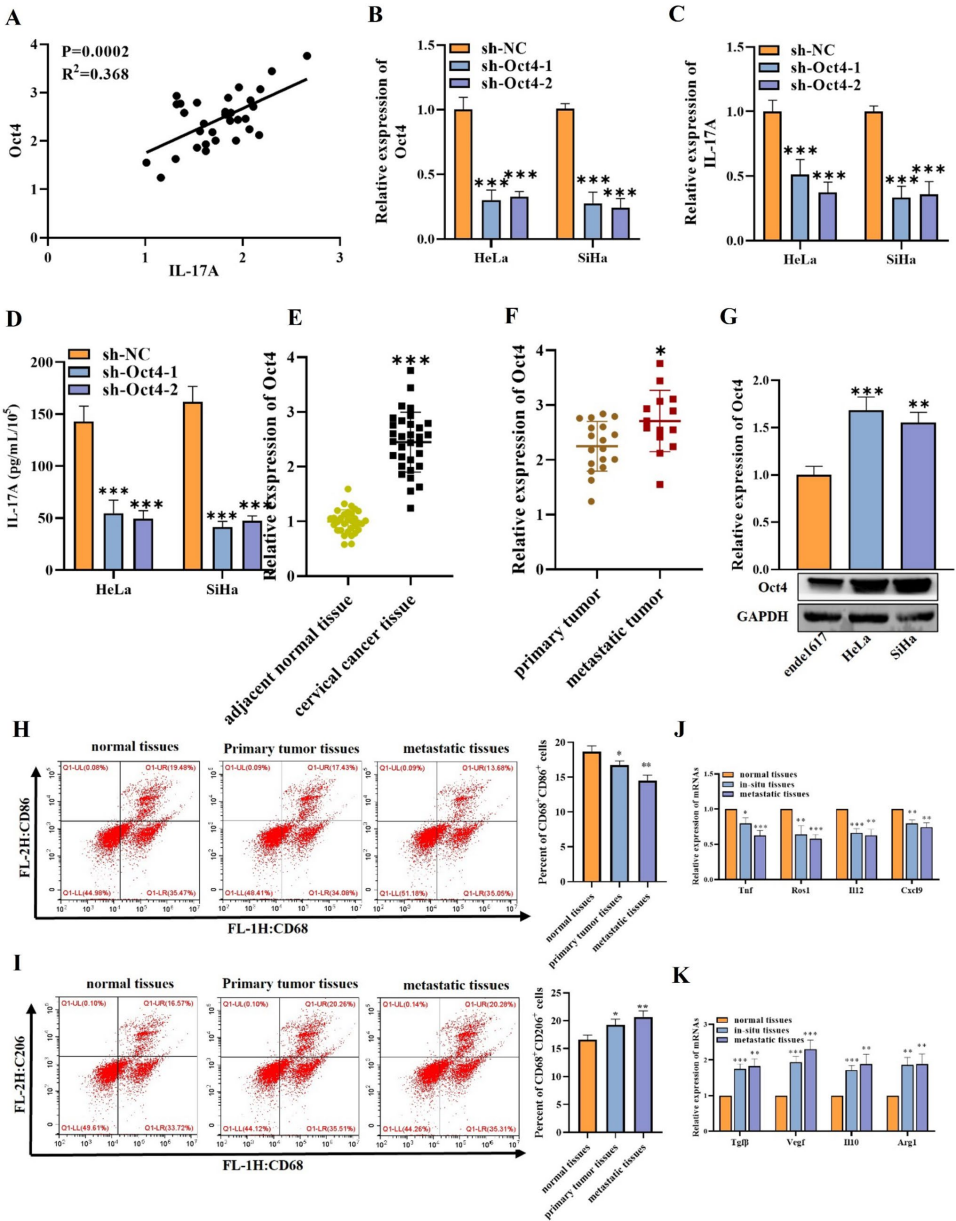
Oct4 promotes the secretion of IL-17A. **A** The correlation between Oct4 and IL-17A in cervical cancer tissues was tested by Spearman correlation analysis. **B**, **C** RT-qPCR outcomes of Oct4 and IL-17A expression in cells transfected with sh-Oct4 or sh-NC. **D** ELISA was utilized to determine IL-17A content in cells transfected with sh-Oct4 or sh-NC. (**E**, **F**) RT-qPCR was used to measure the Oct4 gene expression level in adjacent normal tissues, cervical cancer tissues, primary tumor tissues, and metastatic tumor tissues. **G** RT-qPCR and western blot were used to evaluate Oct4 expression in indicated cells. **H** Surface expression of CD86 was detected in normal tissues, primary tumor tissues, and metastatic tumor tissues using flow cytometry. The histogram represents the percent of CD86 + cells in indicated groups. **I** Surface expression of CD206 was detected in normal tissues, primary tumor tissues, and metastatic tumor tissues using flow cytometry. The histogram represents the percent of CD206^+^ cells in indicated groups. **J** RT-qPCR was utilized to measure the gene expression levels of M1 macrophage markers (Tnf1, Ros1, Il12 and Cxcl9) in indicated groups. **K** RT-qPCR was utilized to measure the gene expression levels of M2 markers (Tgfβ, Vegf, Il10, and Arg1) in indicated groups. ^*^p < 0.05; ^**^p < 0.01; ^***^p < 0.001. All experiments were repeated at least three times

Research shows that octamer-binding transcription factor 4 (OCT4) can induce macrophage polarization to reshape TME [21]. We therefore estimated the correlation between Oct4 and IL-17A in cervical cancer patients. Upon analysis, we found a positive correlation between them (Fig. 3A). We then evaluated the effect of Oct4 inhibition on IL-17A expression. We silenced Oct4 in HeLa and SiHa cells by transfecting with sh-Oct4. RT-qPCR outcomes showed the decrease of Oct4 expression in cells after transfection (Fig. 3B). Later, we measured the impacts of Oct4 silencing on IL-17A expression. As evidenced by RT-qPCR and ELISA, we demonstrated that Oct4 knockdown can reduce IL-17A expression in cancer cells (Fig. 3C, D), suggesting that Oct4 regulates the expression and production of IL-17A in cervical cancer. Based on this, we next explored the expression level of Oct4 in normal and cervical cancer tissues. Upon investigation, we noticed that Oct4 was strongly expressed in cervical cancer tissues, metastatic tissues, HeLa, and SiHa cells (Fig. 3E–G). In addition, studies have demonstrated in vivo and in vitro that IL-17A can induce M2 macrophage in endometriosis [19, 22]. To confirm whether IL17 in human cervical cancer cells plays the same role for macrophages as it does in vitro cell lines, we quantified M1 and M2 macrophage populations in normal tissues, primary tumor tissues, and metastatic tissues of cervical cancer with high IL-17A expression using flow cytometry and showed significantly increased CD206 cells and decreased CD86 cells in cervical cancer cells (Fig. 3H, I**)**. To further confirm the phenotype of these macrophages, the gene expression of typical M1 markers (Tnf, Ros1, Il12, and Cxcl9) and M2 markers (Tgfβ, Vegf, Il10, and Arg1) was investigated in cervical cancer cells and normal adjacent tissue, which presented similar results to that of the flow cytometry data (Fig. 3J, K). Collectively, these data indicated that Oct4 overexpression can promote IL-17A levels in cervical cancer.

### Oct4 promotes cell malignant phenotype in cervical cancer via activating p38 pathway and polarizing M2 macrophage

**Fig. 4:**
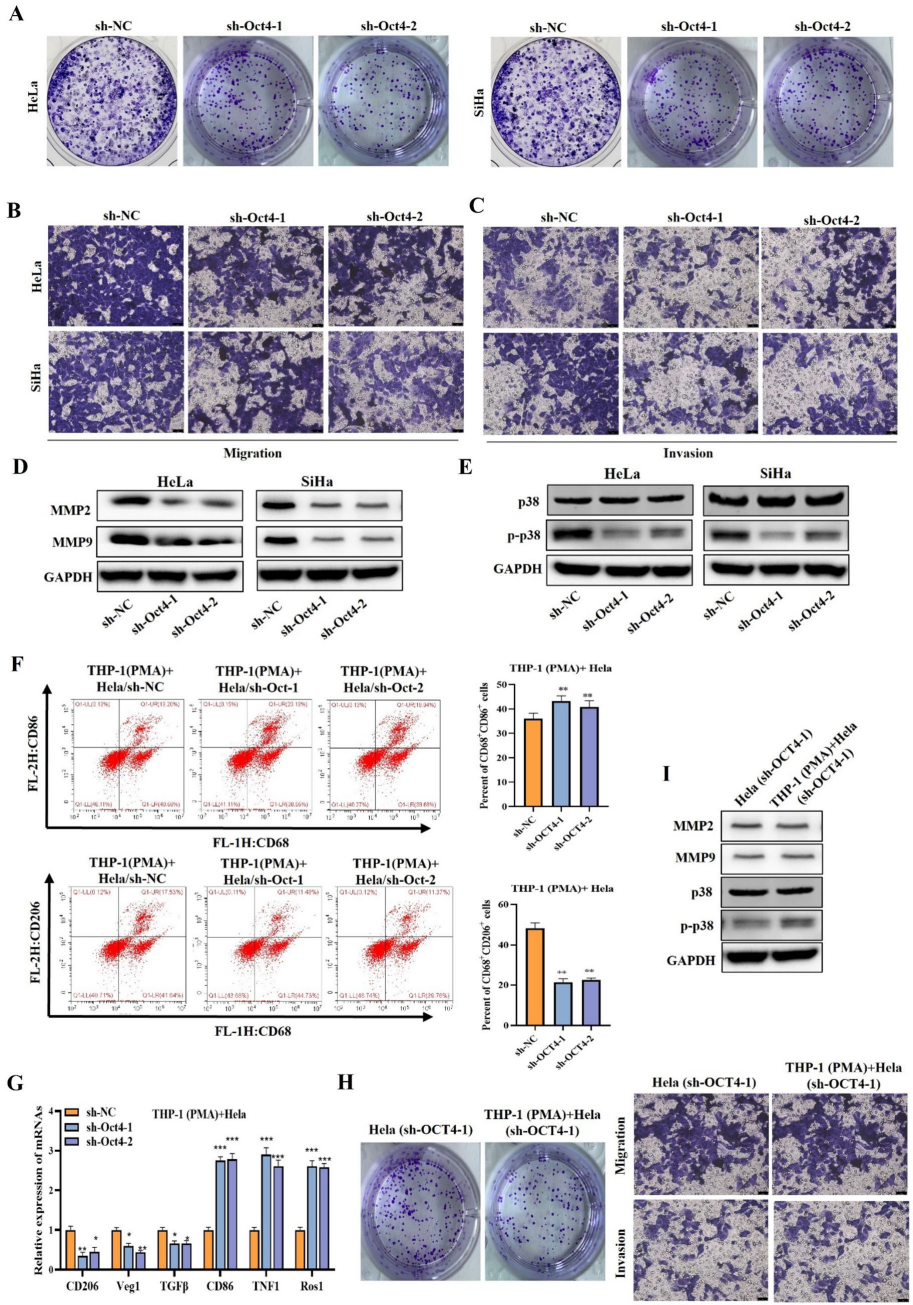
Oct4 promotes cell malignant phenotype and M2 macrophage polarization in cervical cancer by activating p38 pathway. (**A**–**C**) Cell proliferative, migratory, and invasive capabilities were evaluated by colony formation **a** and transwell assays **b–c** when Oct4 was silenced. **D**, **E** Western blot was used to measure MMP2, MMP9, and p-p38 protein levels in cells when Oct4 was silenced. **F** Flow cytometry was utilized to analyze the frequency of M1 or M2 macrophages. **G** RT-qPCR outcomes of CD206, CD86, Tnf1, Ros1, Tgfβ and Vegf expression in cells when Oct4 was silenced. **H** Cell proliferative and migratory capabilities were evaluated by colony formation and transwell assays in groups of Hela (sh-OCT4-1) and THP-1(PMA) + sh-OCT4. **I** Western blot was used to measure MMP2, MMP9, and p-p38 protein levels in groups of Hela (sh-OCT4-1) and THP-1 (PMA) + sh-OCT4 cells. ^**^p < 0.01; ^***^p < 0.001. All experiments were repeated at least three times

We further tested the impacts of Oct4 on the malignant phenotypes of cervical cancer cells and M2 macrophage polarization. The colony formation assay confirmed that Oct4 silencing markedly suppressed cell proliferation (Fig. 4A). Transwell assay showed that Oct4 knockdown notably weakened the capability of cell migration and invasion (Fig. 4B, C). Studies have confirmed that the p38 signaling pathway is activated in various cancers that promotes the malignant behavior [23]. MMP2 and MMP9 are closely associated with the metastasis of malignant tumor cells [24]. Based on this, we hypothesize that Oct4 might promote cervical cancer malignant behavior via regulating MMP2 and 9 and activating the p38 signaling pathway. Western blot indicated that knocking down Oct4 reduced MMP2 and MMP9 levels in cells and inhibited p-p38 levels, indicating that Oct4 activated the p38 pathway (Fig. 4D, E). These results suggested that Oct4 may promote cervical cancer cell proliferative, migratory, and invasive capabilities by activating the p38 pathway.

Next, through the THP-1(PMA) + Hela(sh-OCT4) co-culture system, we examined the impact of Oct4 on macrophage polarization. We discovered that Oct4 deficiency notably suppressed M2 markers (Tgfβ, Vegf, Il10, and Arg1) expression, and increased M1 markers (Tnf, Ros1, Il12 and Cxcl9) expression. Flow cytometry analysis further verified that Oct4 deficiency increased M1 macrophage proportion and decreased M2 macrophage proportion (Fig. 4F, G**)**. The colony formation assay showed that the THP-1(PMA) + Hela(sh-OCT4) group slowed cell proliferation significantly compared with the Hela(sh-OCT4) group. Transwell assay showed that the THP-1(PMA) + Hela(sh-OCT4) group weakened the capability of cell migration and invasion more than the Hela(sh-OCT4) group (Fig. 4H). MMP2, MMP9 and p-p38 expression showed the same level of decline in the group of THP-1(PMA) + sh-OCT4 cells (Fig. 4I). Taken together, these results confirmed that Oct4 promotes cervical cancer cell proliferative, migratory, and invasive capabilities by activating the p38 pathway and polarizing M2 macrophage.

### Oct4 activates IL-17A and affects p38 pathway by binding to IL-17A promoter

**Fig. 5:**
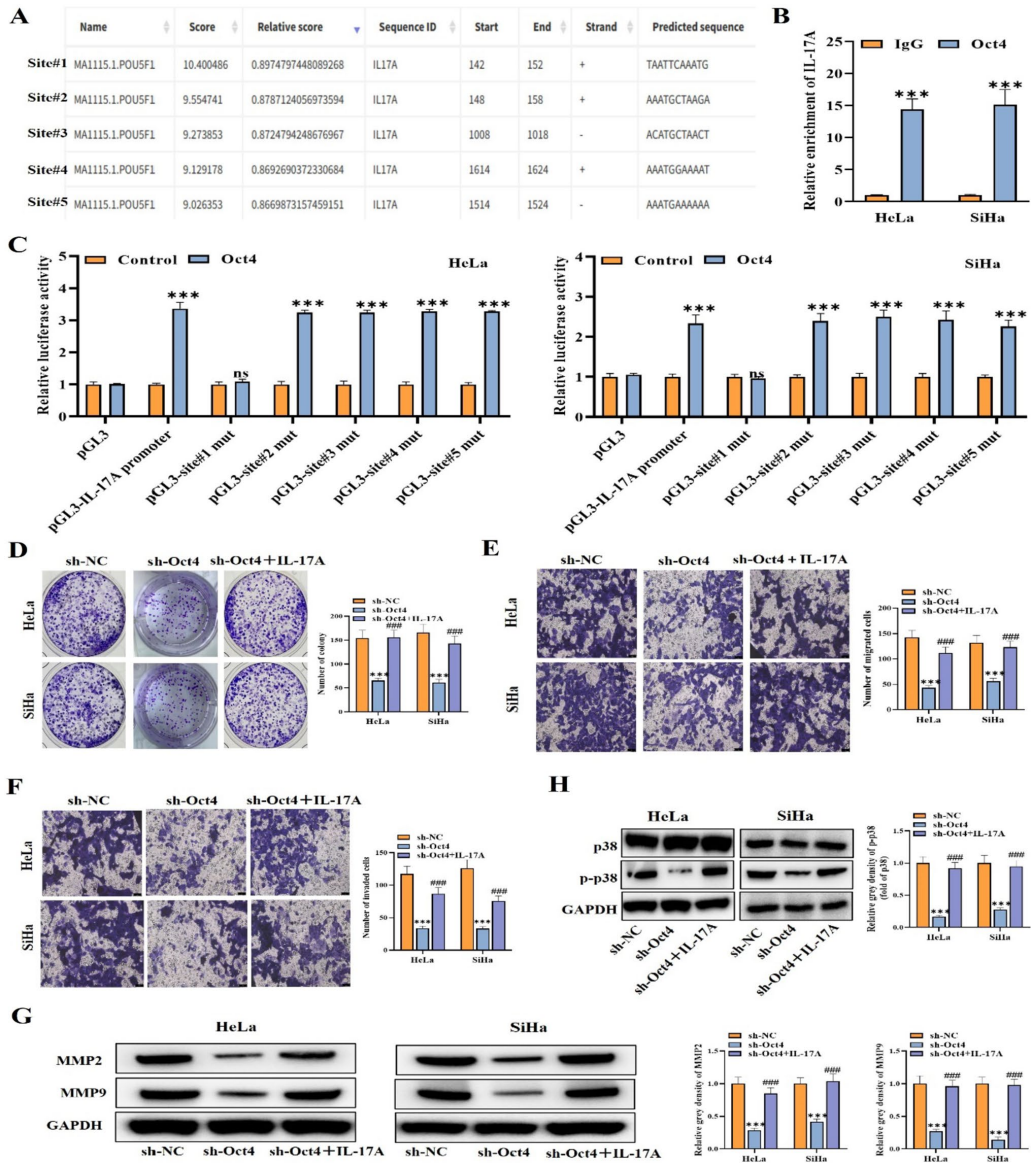
Oct4 activates IL-17A by binding to IL-17A promoter by activating p38 pathway. **A** JASPAR was utilized to predict the possible binding sites of Oct4 and IL-17A promoter. **B**, **C** ChIP assay and luciferase reporter assay were employed for verifying the interplay of Oct4 and IL-17A promoter. **D**–**F** Cell proliferation, migration, and invasion were assessed by colony formation **d** and transwell assays **e**–**f** when Oct4 was silenced, and IL-17A was added. **G**, **H** Western blot outcomes of MMP2, MMP9, and p-p38 protein levels in different cell groups. ^***^p < 0.001; ns means no significance. All experiments were repeated at least three times

The regulatory mechanism between Oct4 and IL-17A in cervical cancer was further estimated. Through JASPAR database, we found five possible binding sites of Oct4 on IL-17A promoter (Fig. 5A). It was indicated by ChIP assay that IL-17A was abundantly enriched by anti-Oct4, suggesting Oct4 can bind to IL-17A promoter (Fig. 5B). Furthermore, luciferase reporter assay was performed and the luciferase activity of IL-17A promoter was markedly increased via upregulating Oct4. However, after site#1 mutation, IL-17A promoter activity was notably reduced, and there was no significant difference with the control group, indicating that site#1 was the site where Oct4 targeted the IL-17A promoter (Fig. 5C). Thus, Oct4 was indicated to transcriptionally activate IL-17A in cervical cancer cells. Thereafter, rescue assays were carried out to verify the regulatory function of Oct4 and IL-17A on cervical cancer cell behaviors. HeLa/SiHa cells transfected with sh-Oct4 or sh-NC were then treated with IL17A to test if IL-17A could mitigate the impact of Oct4 knockdown. As evidenced by colony formation assay and transwell assays, we found that cell proliferative, migratory, and invasive capabilities reduced by Oct4 knockdown were markedly reversed by IL-17A addition (Fig. 5D–F). Furthermore, western blot illustrated that MMP2, MMP9, and p-p38 protein levels decreased by Oct4 downregulation were recovered by IL-17A addition (Fig. 5G, H). These results confirmed that Oct4 can transcriptionally activate IL-17A, which then affects the p38 pathway and regulates the development of cervical cancer.

### Oct4 promotes tumor growth, metastasis, and M2 macrophage infiltration of cervical cancer

**Fig. 6:**
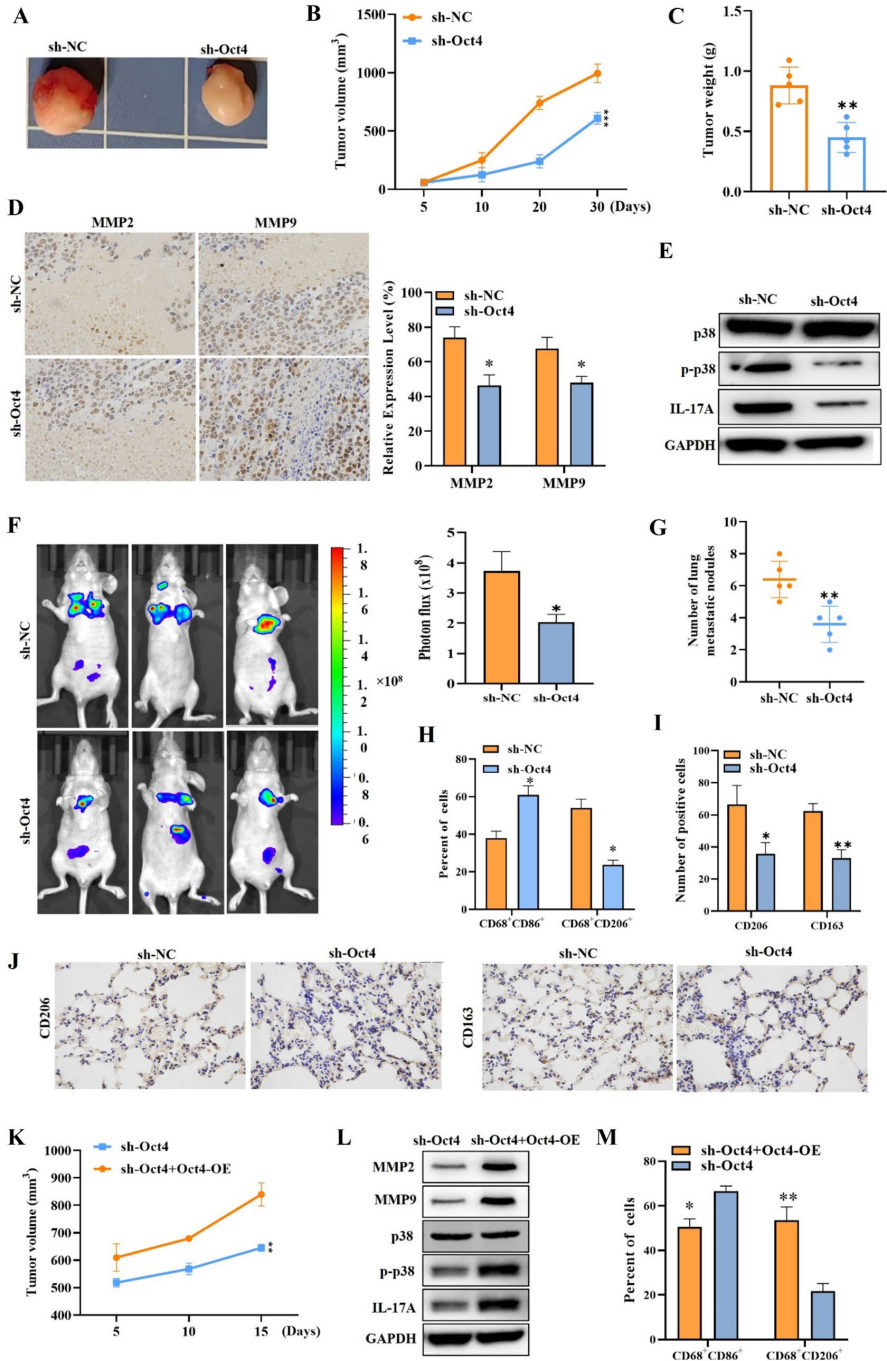
Oct4 promotes tumor growth, metastasis, and M2 macrophage infiltration of cervical cancer*.* **A**–**C** Tumor morphology, volume, and weight of mice injected with HeLa cells transfected with sh-Oct4 or sh-NC. **D** IHC staining of MMP2 and MMP9 in tumor tissues of different groups. **E** Western blot outcomes of IL-17A and p-p38 protein levels in mice tumor injected with HeLa cells transfected with sh-Oct4 or sh-NC. **F** Representative bioluminescence images and the histogram showing the lung metastatic lesions of mice. **G** Pulmonary metastatic nodules were calculated by manually counting individual lesions from metastatic tissue sections in different groups of mice. **H** The percent of CD86^+^ or CD206^+^ cells were assessed by flow cytometry. **I** The expression of CD206 and CD163 levels in mice was measured using IHC. **J** IHC staining was utilized for assessing the quantity of CD206-positive cells and CD163-positive cells in lung metastases. **K** Mice tumor volume of the sh-Oct4 + OCT4-OE group and the sh-Oct4 group. **L** Western blot results of MMP2, MMP9, and p-p38 expression in the tumor tissues of the sh-Oct4 + OCT4-OE group and the sh-Oct4 group. **M** Flow cytometry was utilized to analyze the frequency of M1 or M2 macrophages in different groups of mice. ^*^p < 0.05; ^**^p < 0.01; ^***^p < 0.001. All experiments were repeated at least three times

Lastly, we also validated the functional role of Oct4 in mediating the malignant behavior of cervical cancer cells and M2 macrophage polarization in vivo. Animal experiments were carried out for further confirming Oct4 function on tumor growth of cervical cancer. As shown in Fig. 6A, the tumors in the sh-Oct4 group notably were smaller than controls. The measurement results manifested that knocking down Oct4 can reduce tumor volume and weight (Fig. 6B, C). IHC results illustrated that MMP2 and MMP9 expression in tumor tissue was suppressed by Oct4 knockdown (Fig. 6D). WB results showed that p-p38 and IL-17A expression in tumor tissue was decreased by Oct4 knockdown (Fig. 6E). Furthermore, we established a lung metastasis model of nude mouse. Through the bioluminescence images, we observed that the lung metastasis was alleviated by Oct4 knockdown (Fig. 6F). Subsequently, HE staining indicated that Oct4 knockdown markedly repressed the quantity of pulmonary metastatic nodules (Fig. 6G). These results all indicated that Oct4 silencing inhibited lung metastasis in mice. Then we tested the impact of Oct4 silencing on macrophage polarization in vivo. Flow cytometry analysis indicated that M1 macrophage population was high and M2 macrophage proportion was decreased by Oct4 knockdown (Fig. 6H). The IHC results further confirmed that Oct4 inhibition can effectively reduce the quantity of CD206-positive and CD163-positive cells in lung metastases (Fig. 6I, J). Furthermore, we overexpressed Oct4 in knocked down mice to further validate the role of Oct4 in tumor progression in vivo. Compared with the sh-Oct4 group, the tumor volume decreased significantly in the sh-Oct4 + OCT4-OE group (Fig. 6K). WB results demonstrated that the expression of MMP2, MMP9, and p-p38 was higher in the tumor tissues of the sh-Oct4 + Oct4-OE group compared to the sh-Oct4 group (Fig. 6L). Flow cytometry analysis showed that the proportion of M1 macrophages decreased, whereas the proportion of M2 macrophages increased after Oct4 overexpression (Fig. 6M). Collectively, these results indicated that Oct4 promotes tumor growth, metastasis, and M2 macrophage infiltration.

## Discussion

Cervical cancer is a common malignant tumor in female gynecology with high incidence rate. However, current treatment strategies cannot effectively improve the prognosis of advanced patients [25]. Therefore, in-depth investigation of cervical cancer pathogenesis is of great significance. In cervical cancer development, the interaction between cancer cells and macrophages can form a TME that conduces to tumor metastasis [26]. TAM have been recognized as crucial factor for poor prognosis and can promote tumorigenesis. In TME, macrophages under stress can transform into M2 polarized macrophages, take part in the modulation of angiogenesis, and trigger the metastasis of cancer cells [6]. Several studies have revealed the association between M2 macrophage polarization and tumor development [27]. For example, Circ-ITGB6 accelerates ovarian cancer cisplatin resistance by inhibiting macrophage M2 polarization [28]. MiR-423-3p suppresses macrophage M2 polarization to inhibit cervical cancer development [29]. In the TME, inflammatory reactions involving inflammatory factors significantly affect the development of tumors [30]. IL-17A is a powerful proinflammatory factor, which can regulate different inflammatory diseases [31], and also has been detected to play the crucial function in the TME [32]. Previous studies have confirmed that IL-17A can accelerate the development of lung cancer [13], colorectal cancer [14], and even cervical cancer [20]. In line with these studies, herein, we also confirmed that IL-17A was upregulated in metastatic cervical cancer tissues and cells. Subsequently, through a co-culture system of cancer cells and macrophages, we found that overexpression of IL-17A can promote M2 macrophage polarization in cervical cancer. Furthermore, we found that IL-17A secreted by cervical cancer cells facilitated macrophages mediating the promotion on cell proliferative, migratory, and invasive capabilities. These findings confirm the carcinogenic properties of IL-17A.

Oct4 is a homeodomain transcription factor of the POU family, which maintain embryonic stem cells state [33]. Oct4 has been confirmed to regulate transcription activity of chromatin modifiers and different RNAs [34]. For example, Oct4 transcriptionally activates the expression of NEAT1 and MALAT1 to accelerate lung cancer progression [35]. In recent years, the overexpression and regulatory function of Oct4 in human cancer cells have been demonstrated [36]. It is reported that Oct4 promotes stemness in head and neck squamous cell carcinoma via modulating PSMC3IP and RAD54L [37]. Oct4 drives EMT process and expedites ovarian cancer development [38]. Furthermore, Oct4 has been confirmed to promote tumorigenesis and suppress cell apoptotic capability in cervical cancer [39]. Oct4 activated by HPV facilitates cervical cancer cell growth via inhibiting p53 expression [40]. Additionally, evidence has verified that Oct4 can induce M2 macrophage polarization to reshape TME in lung cancer [21]. However, the relationship between Oct4 and IL-17A in cervical cancer has not yet been investigated. Herein, we confirmed that Oct4 was notably overexpressed in cervical cancer. Oct4 promoted cell proliferation, migration, and invasion, as well as M2 macrophage polarization, confirming the carcinogenic effects of Oct4 in cervical cancer, which is in agreement with the results of above-mentioned studies. Next, the significant positive regulation between Oct4 and IL-17A in cervical cancer was confirmed. Further experiments confirmed that Oct4 directly bound to the IL-17A promoter and transcriptionally activated IL-17A. IL-17A overexpression reversed the suppressive functions of Oct4 knockdown on cell malignant phenotype. The animal experiments further verified that Oct4 facilitated tumor growth and metastasis in mice and increased M2 macrophage infiltration.

Research has shown that the activation of p38 signaling can promote cancer development through interactions with TME [41]. MMP2 and MMP9 are often overexpressed in cancers and promote tumor metastasis [42]. Evidence suggests that p38 activation can upregulate MMP2 and MMP9 [43]. Similarly, we also discovered that Oct4 promoted p38 phosphorylation level and MMP2 and MMP9 levels in cervical cancer cells, indicating the activation of the p38 pathway. Furthermore, we illustrated that MMP2, MMP9, and p-p38 protein levels decreased by Oct4 downregulation were recovered by IL-17A addition. We found that Oct4 directly promotes the capacity of cell proliferation, migration, and invasion after knocking down Oct4 in HeLa/siHa cells. Interestingly, this effect was more pronounced after THP-1(PMA) cells were co-cultured with Oct4 knockdown cells. Therefore, we hypothesize that Oct4 can regulate cervical cancer development by binding IL-17A to modulate p38 pathway, and that Oct4 transcriptionally activates IL-17A to orchestrate M2 macrophage polarization and cervical cancer metastasis. 

## Conclusion

Taken together, this study confirmed that Oct4 transcriptionally activates IL-17A to regulate the p38 signaling pathway and promotes M2 macrophage polarization thereby promoting cervical cancer metastasis. These new findings may offer the novel therapeutic target for treating cervical cancer.

### Supplementary Information

Below is the link to the electronic supplementary material.

Supplementary file1 (DOCX 198 KB)

## Data Availability

The datasets generated during and/or analyzed during the current study are available from the corresponding author upon reasonable request.
