# Supplementary material for: Oct4 activates IL-17A to orchestrate M2 macrophage polarization and cervical cancer metastasis
Source: Cancer Immunol Immunother. 2024 Mar 2;73(4):73. doi: 10.1007/s00262-023-03596-z (PMC10908604; doi:10.1007/s00262-023-03596-z)
Supplement: Supplementary file 1 — Supplementary file1 (DOCX 198 KB) [file 262_2023_3596_MOESM1_ESM.docx]

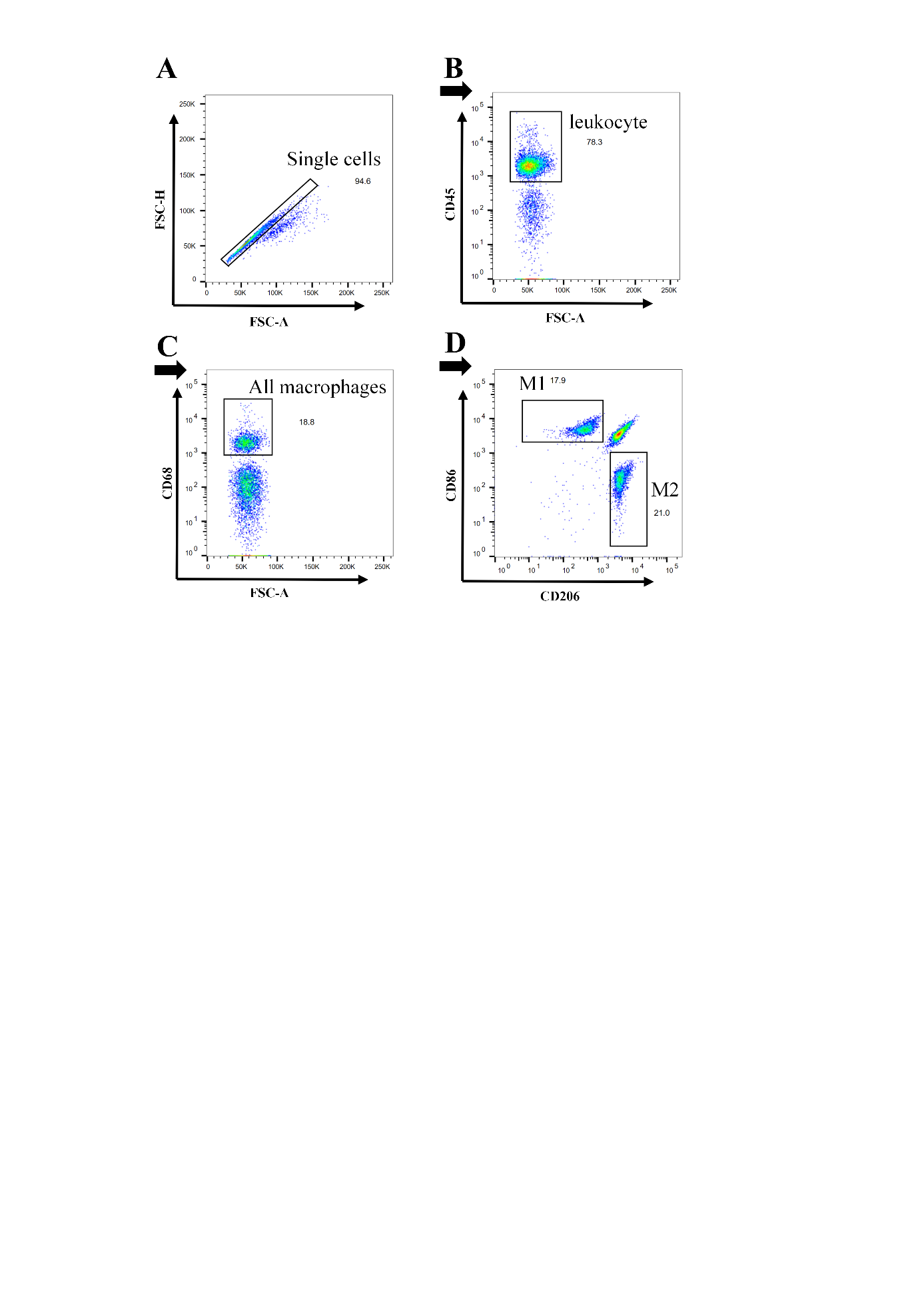


**Figure S1. Gating strategy to identify M1 and M2 macrophage populations.** (A) Single cells were gated using FSC. (B) Leukocytes were identified using CD45+. (C) Within the leukocyte population, all macrophages (CD68+) were distinguished. (D) Differentiation between M1 (CD86+CD206−) and M2 macrophages (CD86-CD206+) was identified.
